# Towards joint segmentation and registration of the myocardium in CP-BOLD MRI at rest

**DOI:** 10.1186/1532-429X-18-S1-W34

**Published:** 2016-01-27

**Authors:** Ilkay Oksuz, Rohan Dharmakumar, Sotirios Tsaftaris

**Affiliations:** 1 IMT Institue for Advanced Studies Lucca, Lucca, Italy; 2 Cedars-Sinai Medical Center, Los Angeles, CA USA; 3 University of Edinburgh, Edinburgh, United Kingdom

## Background

A new contrast and stress-free approach for detecting myocardial ischemia has been recently demonstrated using Cardiac Phase-resolved Blood Oxygen-Level-Dependent (CP-BOLD) MRI. To detect the disease, CP-BOLD relies on the observation that myocardial signal intensity varies as a function of cardiac phase. These changes are not readily visible and post-processing is necessary; including myocardial segmentation and registration. Automated analysis approaches, which can obtain pixel-level determination of ischemia, are desirable since they may lead to improved accuracy in detection of disease. To achieve this, precise segmentation and non-linear registration of the myocardium among the frames (the cardiac phases) in the cine stack would be required. Unfortunately, at present due to BOLD contrast variations, classical approaches to segmentation and registration fail to reach desirable accuracy. Here we propose an algorithm that jointly finds suitable myocardial segmentation and elastically registers the heart.

## Methods

**Figure 1:**
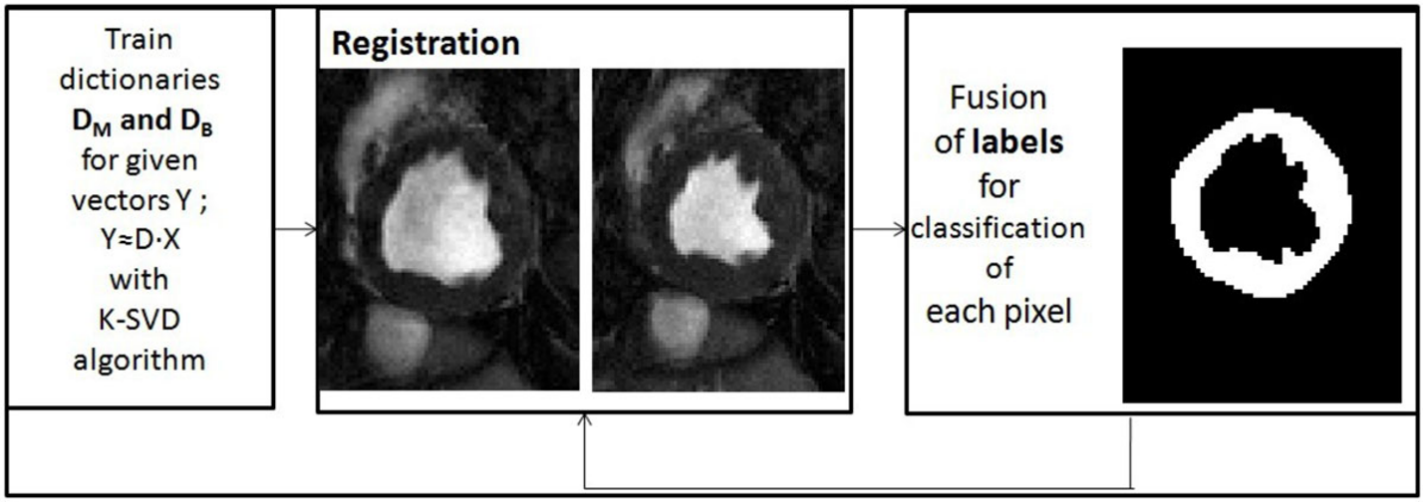
**The figure at top shows the flowchart of the algorithm**. First 9 × 9 patches are extracted for each pixel and concatenated with HOG and Gabor features of each patch. Dictionaries D_B_ and D_M_ are calculated for the initialization of registration process. The sparse representation of each pixel p for background X_B_ and myocardium X_M_ are calculated and concatenated; X_p_ =[X_B_, X_M_ ]. In data term of the objective function of registration; the similarity metric in between two consecutive frames is defined as L1 norm of the concatenated supports: S(I_t_(p), I_t+1_(p+u)) = ||X^t^_p_-X^t+1^_p+u_||_1_. The approach iterates refinement step of the segmentation using the registration for the agreement of classification and then finding the D, then X. The fusion of labels in between the different frames is used for classification of the myocardium region.

Flow- and motion-compensated 2D short-axis CP-BOLD was acquired along the mid ventricle in 10 canines at baseline and under severe LAD stenosis. Imaging studies were performed on a 1.5T Espree (Siemens Healthcare). TR/TE 6.2/3.1 ms; spatial resolution = 1.2 × 1.2 × 8 mm3, flip-angle = 70°, ~25 frames per cardiac cycle. The main principle of the proposed approach is to use an external initial database of pre-segmented images (and dictionaries for myocardium and background), to first come up with an initial segmentation (relying on classification when projecting on discriminatory dictionaries) of an input CP-BOLD patient stack. Then, images in the cardiac cycle (cine acquisition) are registered using the sparse coefficients of the projections to establish a new similarity metric. Afterwards, the obtained segmentation is refined; which gives the opportunity to refie the dictionaries (adapting them to the dataset under consideration). This refinement of segmentation and dictionaries is continued till convergence. The flowchart and detailed explanation of the algorithm is shown in figure 1.

## Results

As seen in table 1, experiments under baseline and ischemia conditions show that the proposed approach improves the results thanks to the refinement with registration. Morever; the proposed approach obtains superior accuracy when compared to state of the art methods for registration (DRAMMS and MIND).

**Table 1:** The values show the average dice scores and standard deviations.

	CP-BOLD BASELINE	CP-BOLD ISCHEMIA
Proposed Approach	0.68 ± 0.08	0.63 ± 0.06
Proposed without refinement	0.66 ± 0.09	0.60 ± 0.13
DRAMMS	0.61 ± 0.07	0.54 ± 0.06
MIND	0.62 ± 0.07	0.53 ± 0.09

## Conclusions

The experiments clearly underline the need for a new representation in image registration and segmentation. Using the information from the same subject to train the dictionaries improves the accuracy of myocardium segmentation both in the cases of health and disease. Although further experiments are necessary to validate this approach, CP-BOLD can open the road to repeatable, truly non-invasive diagnosis of ischemic heart disease.

Dice overlap measures for the proposed method compared with segmentation without refinement and two state of the art methods for image registration DRAMMS (Ou et al., MIA, 2011) and MIND (Heinrich et al., MIA, 2012)

